# Supplementary material for: Assessment of Data-Independent Acquisition Mass Spectrometry (DIA-MS) for the Identification of Single Amino Acid Variants
Source: Proteomes. 2024 Nov 6;12(4):33. doi: 10.3390/proteomes12040033 (PMC11587465; doi:10.3390/proteomes12040033)
Supplement: Supplementary file 1 [file proteomes-12-00033-s001.zip › proteomes-3201495-supplementary.pdf]

Supplementary Material

Assessment of data-independent acquisition mass spectrometry (DIA-MS) for the identification of single amino acid variants.

Ivo Fierro-Monti 1,2*, Klemens Fröhlich 2, Christian Schori 2, and Alexander Schmidt 2*

1 EMBL – EBI, Hinxton, Cambridgeshire, United Kingdom

2 University of Basel, Biozentrum, Basel, Switzerland

*Correspondence: alex.schmidt@unibas.ch

Figure S1:

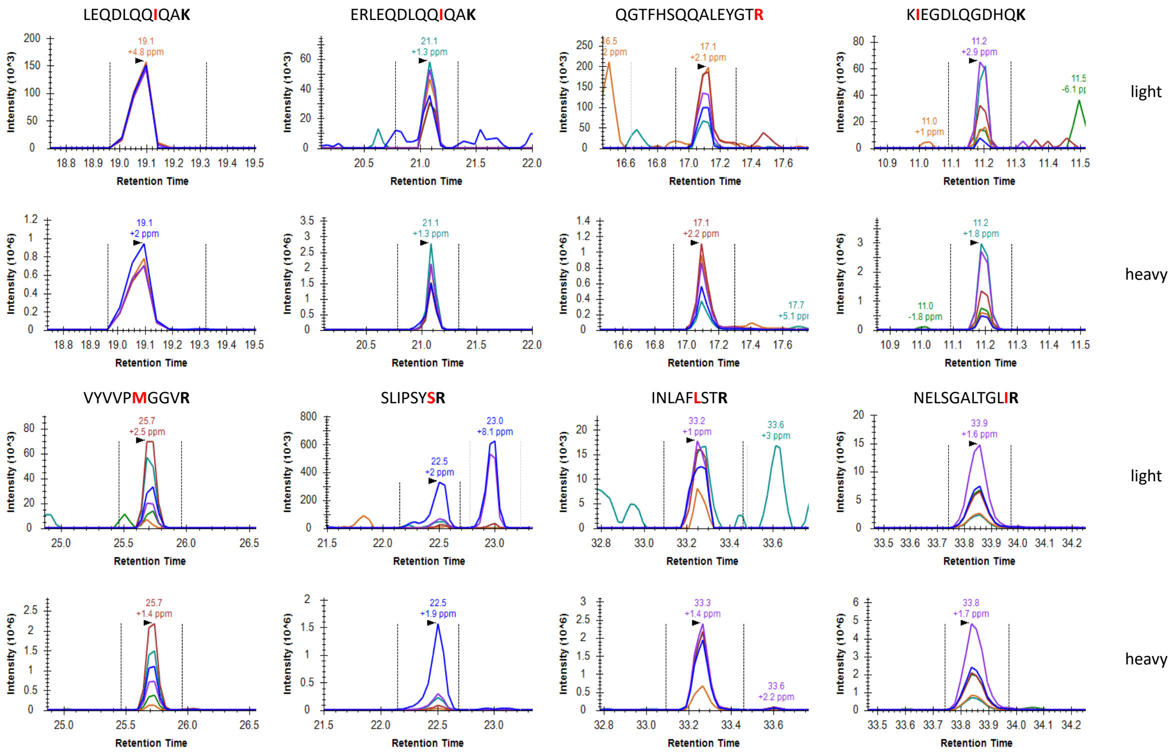

Figure S1: Variant peptide fragment ion chromatograms representing the intensities of each of the corresponding fragments (manually curated based on stable isotope labelled spike-in peptide standards) versus the Retention time. Here, the peptide fragment ion chromatograms of the (heavy) reference and the corresponding (light) SAAV peptide are shown.

Figure S2:

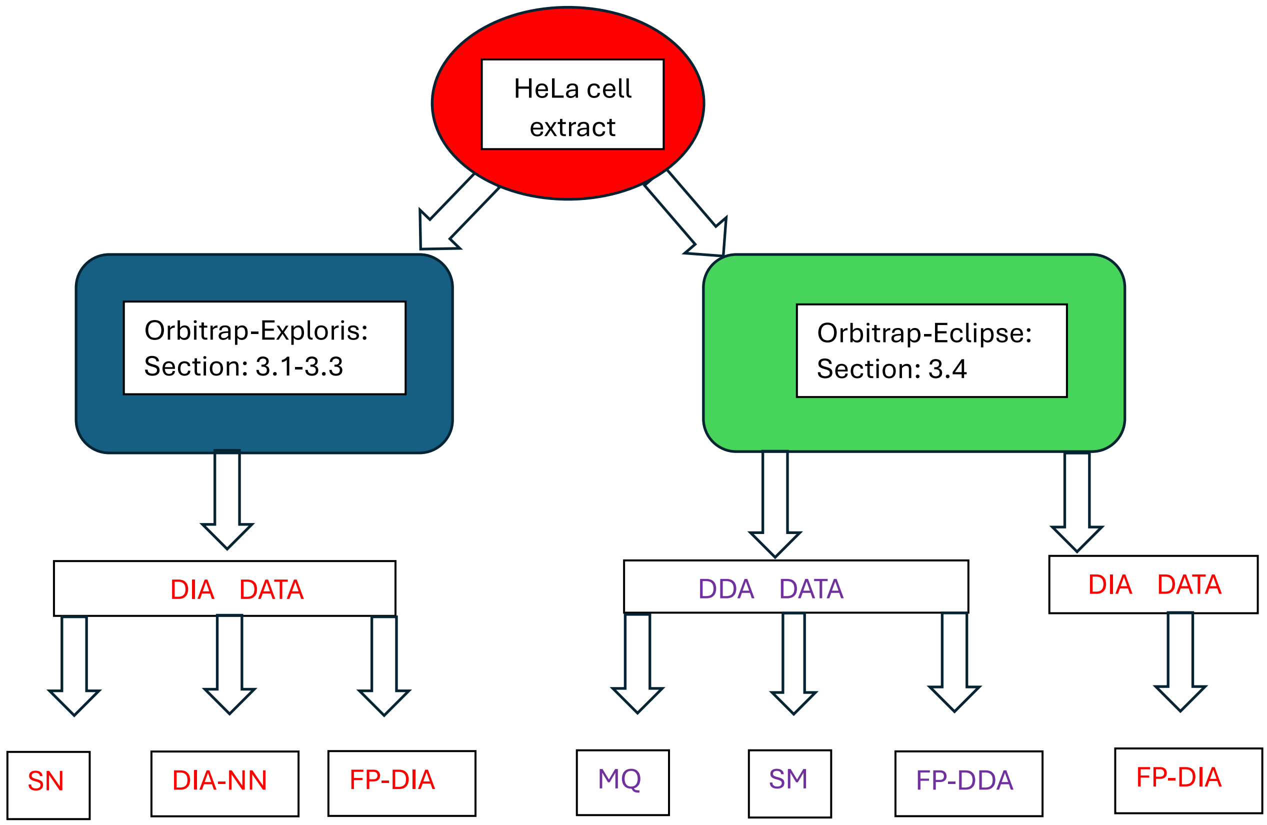

	Spectronaut _SN_output: **DIA_SN_ search_output_ Orbi_Exploris_ data.zip**	DIA-NN output: **DIA_DIA-NN_search_output_Orbi_Exploris _data.zip**	Fragpipe_DIA_ FP-DIA_output: **DIA_FP-DIA_search_output_Orbi_Exploris_data.zip**	MaxQuant MQ_output: **DDA_MQ_ search_output_ Orbi_Eclipse_ data.zip**	Spectromine SM_output: **DDA_SM_search _output_Orbi_ Eclipse _data.zip**	FragpipeDDA FP-DDA output: **DDA_FP-DDA_search_output_Orbi_ Eclipse _data.zip**	FragpipeDIA FP-DIA output: **DIA_FP-DIA_search_output_Orbi_ Eclipse _data.zip**
SearchDB_ used:	Output files in zip:	Output files in zip:	Output files in zip:	Output files in zip:	Output files in zip:	Output files in zip:	Output file in zip:
CPS_DB	SN_CPS_DB	DIANN_CPS_DB	FP-DIA_CPS_DB	MQ_CPS_DB	SM_CPS_DB	FP-DDA_CPS_DB	FP-DIA_CPS_DB
1x decoyDB	SN_1x_DB	DIANN_1xDB	FP-DIA_1xDB	MQ_1x_DB	SM_1x_DB	FP-DDA_1x_DB	na
10x decoyDB	na	na	na	MQ_10x_DB	SM_10x_DB	FP-DDA_10x_DB	na
100x decoyDB	SN_100x_DB	DIANN_100xDB	FP-DIA_100xDB	MQ_100x_DB	SM_100x_DB	FP-DDA_100x_DB	na
400x decoyDB	SN_400x_DB	DIANN_400xDB	FP-DIA_400xDB	MQ_400x_DB	SM_400x_DB	FP-DDA_400x_DB	na
Pyf decoyDB	SN_Pyr_DB	DIANN_Pyr DB	FP-DIA_PyrDB	MQ_Pyr_DB	SM_Pyr_DB	FP-DDA_Pyr_DB	na
List of Supplementary pipeline's DIA and DDA output files from Orbitrap Eclipse and Orbitrap Exploris instruments:							
All files are available via MassIVE using the following link: ftp://MSV000095082@massive.ucsd.edu							

Figure S2: Overview of the database search results obtained from the different MS raw files and software pipelines used.
